# Signals of adaptation to agricultural stress in the genomes of two European bumblebees

**DOI:** 10.3389/fgene.2022.993416

**Published:** 2022-10-05

**Authors:** Alex F. Hart, Jaro Verbeeck, Daniel Ariza, Diego Cejas, Guillaume Ghisbain, Hanna Honchar, Vladimir G. Radchenko, Jakub Straka, Toshko Ljubomirov, Thomas Lecocq, Juliana Dániel-Ferreira, Simone Flaminio, Laura Bortolotti, Reet Karise, Ivan Meeus, Guy Smagghe, Nicolas Vereecken, Peter Vandamme, Denis Michez, Kevin Maebe

**Affiliations:** ^1^ Ghent University, Faculty of Bioscience Engineering, Department of Plants and Crops, Lab of Agrozoology, Ghent, Belgium; ^2^ Laboratory of Zoology, Research Institute for Biosciences, University of Mons, Mons, Belgium; ^3^ Smithsonian Tropical Research Institute, Gamboa, Panama; ^4^ Institute for Evolutionary Ecology, National Academy of Sciences of Ukraine, Kyiv, Ukraine; ^5^ Charles University, Faculty of Science, Department of Zoology, Praha, Czech Republic; ^6^ Institute of Biodiversity and Ecosystem Research—Bulgarian Academy of Sciences, Sofia, Bulgaria; ^7^ Université de Lorraine, INRAE, URAFPA, Nancy, France; ^8^ Department of Ecology, Swedish University of Agricultural Sciences, Uppsala, Sweden; ^9^ Council for Agricultural Research and Economics, Research Centre for Agriculture and Environment, Bologna, Italy; ^10^ Estonian University of Life Sciences, Institute of Agricultural and Environmental Sciences, Tartu, Estonia; ^11^ Agroecology Lab, Université Libre de Bruxelles (ULB), Brussels, Belgium; ^12^ Laboratory of Microbiology, Department of Biochemistry and Microbiology, Ghent University, Ghent, Belgium

**Keywords:** *Bombus*, global change, RADseq, population genomics, agricultural intensification, bee decline, Anthropocene

## Abstract

Human-induced environmental impacts on wildlife are widespread, causing major biodiversity losses. One major threat is agricultural intensification, typically characterised by large areas of monoculture, mechanical tillage, and the use of agrochemicals. Intensification leads to the fragmentation and loss of natural habitats, native vegetation, and nesting and breeding sites. Understanding the adaptability of insects to these changing environmental conditions is critical to predicting their survival. Bumblebees, key pollinators of wild and cultivated plants, are used as model species to assess insect adaptation to anthropogenic stressors. We investigated the effects of agricultural pressures on two common European bumblebees, *Bombus pascuorum* and *B. lapidarius*. Restriction-site Associated DNA Sequencing was used to identify loci under selective pressure across agricultural-natural gradients over 97 locations in Europe. 191 unique loci in *B. pascuorum* and 260 in *B. lapidarius* were identified as under selective pressure, and associated with agricultural stressors. Further investigation suggested several candidate proteins including several neurodevelopment, muscle, and detoxification proteins, but these have yet to be validated. These results provide insights into agriculture as a stressor for bumblebees, and signal for conservation action in light of ongoing anthropogenic changes.

## Introduction

Humans have been transforming their surrounding landscapes, the oceans, and the atmosphere, in a time period known as the Anthropocene (Crutzen and Stoermer, 2000; Ruddiman, 2013; Lewis and Maslin, 2015). This epoch is characterised by an increase in atmospheric greenhouse gases, changes in land surface structure and composition *via* deforestation, urbanisation, industrial and agricultural development, and a massive reduction in global biodiversity (e.g., Dirzo et al., 2014; Lewis and Maslin, 2015; Sage, 2020). Insects are not immune to the pressures of the Anthropocene, and are also suffering major declines in both abundance and diversity (e.g., Sánchez-Bayo and Wyckhuys, 2019; Wagner et al., 2021). Considerable habitat losses due to deforestation, urban development, intensive agriculture, and changing climatic conditions that are often largely different from the environmental conditions to which they are inherently adapted, are examples of anthropogenic threats (e.g., Sánchez-Bayo and Wyckhuys, 2019; Wagner et al., 2021). In Europe, agricultural practices were identified as a major driver of negative population trends (Van Swaay and Warren, 2012; Nieto et al., 2014).

The wide range of effects of modern intensive agriculture on insect populations have been well-studied (Raven & Wagner, 2021), especially in the contexts of agrochemicals and crop pests (reviewed in Sánchez-Bayo, 2011; Raven and Wagner, 2021; Gunstone et al., 2021). The impact of agricultural practices on insect populations is mostly studied using indirect approaches, studying the by-effects of agricultural intensification and subsequent habitat fragmentation and habitat loss, rather than a direct approach, studying how conspecific populations develop in both natural and agricultural environments (Goulson et al., 2005; Kosior et al., 2007; Goulson et al., 2008). However, direct approaches have already been used to: 1) identify genetic differentiation between populations, associated with habitat differences (Hereward et al., 2013; Andrews et al., 2020); for example, the hemipteran agricultural pest *Creoniades dilutes* shows local adaptation to natural arid environments and agricultural sites, thought to be linked to host plant specialisation (Hereward et al., 2013). Similarly, Andrews et al. (2020) found evidence of adaptation to agricultural environments in populations of Elaterid beetles of the genus *Limonius* which was likely correlated with pesticide regimes; 2) or even adaptive responses to insecticide exposure; for instance in the Colorado potato beetle *Leptinotarsa decemlineata* and mosquito *Anopheles arabiensis*, which show significant upregulation of detoxifying cytochrome P450s after agrochemical exposure (Zhu et al., 2016; Oliver and Brooke, 2018), and in *Spodoptera exigua* and *Nilaparvata lugens* which show upregulation of Heat Shock Protein 70 in response to insecticides (You et al., 2016; Tarnawska et al., 2019).

Bumblebees are considered a model species to study anthropogenic influence on insects (Gérard et al., 2021; Maebe et al., 2021; Martinet et al., 2021; Theodorou et al., 2021). Both managed and wild bumblebee species are economically important keystone pollinators of mostly temperate climates, and have relatively generalist diets (Velthuis and Van Doorn, 2006; Klein et al., 2007, but see Wood et al., 2021). A significant proportion of *Bombus* species show declining population trends (Cameron and Sadd, 2020; Rasmont et al., 2021), while a few remain stable or are currently expanding their distribution range (Biella et al., 2020; Ghisbain et al., 2021).

The decline of bumblebee populations has been especially well-studied in Europe and North-America (e.g. Cameron et al., 2011; Kerr et al., 2015; Rasmont et al., 2015; Cameron & Sadd, 2020), and although declines are caused by many interacting factors (Williams and Osborne, 2009), they are mainly attributed to agricultural intensification, especially the loss and fragmentation of natural habitats (Kosior et al., 2007; Goulson et al., 2008; Marshall et al., 2018; Vray et al., 2019). A diverse range of biotic and abiotic stressors impacting bumblebees have been associated with agricultural intensification. Pesticides, such as neonicotinoids, are known to negatively impact bumblebee populations through mortality and sublethal effects (Thompson, 2001; Goulson et al., 2015; Anderson and Harmon-Threatt, 2019). Intensive agriculture is also known to reduce nesting site availability, both directly *via* the removal of hollows such as trees and leaf litter, and indirectly *via* a reduction in rodent burrow abundance (Alford, 1975; Goulson et al., 2008; Liczner and Colla, 2019). Agricultural conditions can generate nutritional stress, providing inadequate nutrient diversity (Vaudo et al., 2020), and/or on a shorter period than the activity period of the species (Goulson et al., 2008; Vanderplanck et al., 2019a). Furthermore, the introduction of non-native or managed bee taxa in agricultural areas can spread both endemic and introduced pathogens to native bee populations (Goulson et al., 2008; Meeus et al., 2011; Graystock et al., 2013; Yañez et al., 2020). Finally, stressors may work multiplicatively to an overall stronger negative effect; for example, pathogen exposure combined with nutritional stress in agricultural environments was found to be a contributor to the regression of bumblebee populations (McNeil et al., 2020).

Although intensive agriculture and habitat fragmentation are a relatively recent phenomenon on an evolutionary scale, recent research has paid a lot of attention to their anthropogenic impact on bumblebees and their resilience to these new stressors (Maebe et al., 2021). Numerous studies have investigated several morphological traits in bumblebees that may be under selective pressure in agricultural environments, and primarily, body size has been associated with species-specific responses to agricultural intensification (Gérard et al., 2020, 2021) and urbanization (Theodorou et al., 2021; Tommasi et al., 2022). However, the increase in body size might be mainly or solely explained by the increased level of habitat fragmentation in urban and agricultural areas (Theodorou et al., 2021 and Gérard et al., 2021, respectively).

The loss and fragmentation of habitats have been shown to negatively affect both European and North American bumblebee species, leading to smaller, more isolated populations with lower genetic diversity due to genetic drift and inbreeding, which causes vulnerability to additional threats (e.g. Goulson et al., 2008; Jha and Kremen, 2013; Jha, 2015). Furthermore, Jha (2015) found that agricultural areas can act as significant barriers to gene flow in bumblebees. By comparing *B. lapidarius* collected from several urban and rural locations in Germany, Theodorou et al. (2018) found evidence of directional selection associated with urban land, identifying genes related to heat stress, oxidative stress, and metabolism as under selective pressure. Agricultural areas are therefore known to act as barriers to gene flow and to cause habitat fragmentation, which in turn can lead to differential population structuring and local adaptation. To date however, no study has taken a direct approach comparing wild bumblebees from natural and agricultural landscapes to explore possible genomic adaptations to the associated stressors.

The aim of this study was to identify genomic regions and/or underlying genes reflecting the different selective pressures on bumblebees between agricultural and natural areas. We used Restriction-site Associated DNA sequencing (henceforth RADSeq) analyses on specimens of two bumblebee species that are widespread and abundant in Europe: *B. pascuorum* (Scopoli, 1763) and *B. lapidarius* (Linnaeus, 1758)*.* RADSeq is a powerful and cost-efficient technique for massive SNP discovery in large populations (Baird et al., 2008; Narum et al., 2013). Such reduced representation approaches allow for thorough coverage of the genome without the high costs associated with whole genome sequencing (WGS) (Baird et al., 2008; Davey et al., 2011; Narum et al., 2013), and are being used to highlight population structure, detect loci under selective pressure, and adaptation, among others, in bumblebees (e.g., Lozier et al., 2016; Jackson et al., 2018, 2020; Theodorou et al., 2018; Silva et al., 2020). Furthermore, we explored and ruled out population differentiation and demographic history as interfering factors when testing for signatures of selection (Nielsen, 2001; but see Theodorou et al., 2018). For this study, we hypothesise that the combination of several agricultural pressures (such as pesticide use, pathogens, and habitat fragmentation) will be detectable in both bumblebee species as a consequence of selection, including in genes related to detoxification, immunity and/or flight muscle development.

## Materials and methods

### Model species and sampling

**FIGURE 1:**
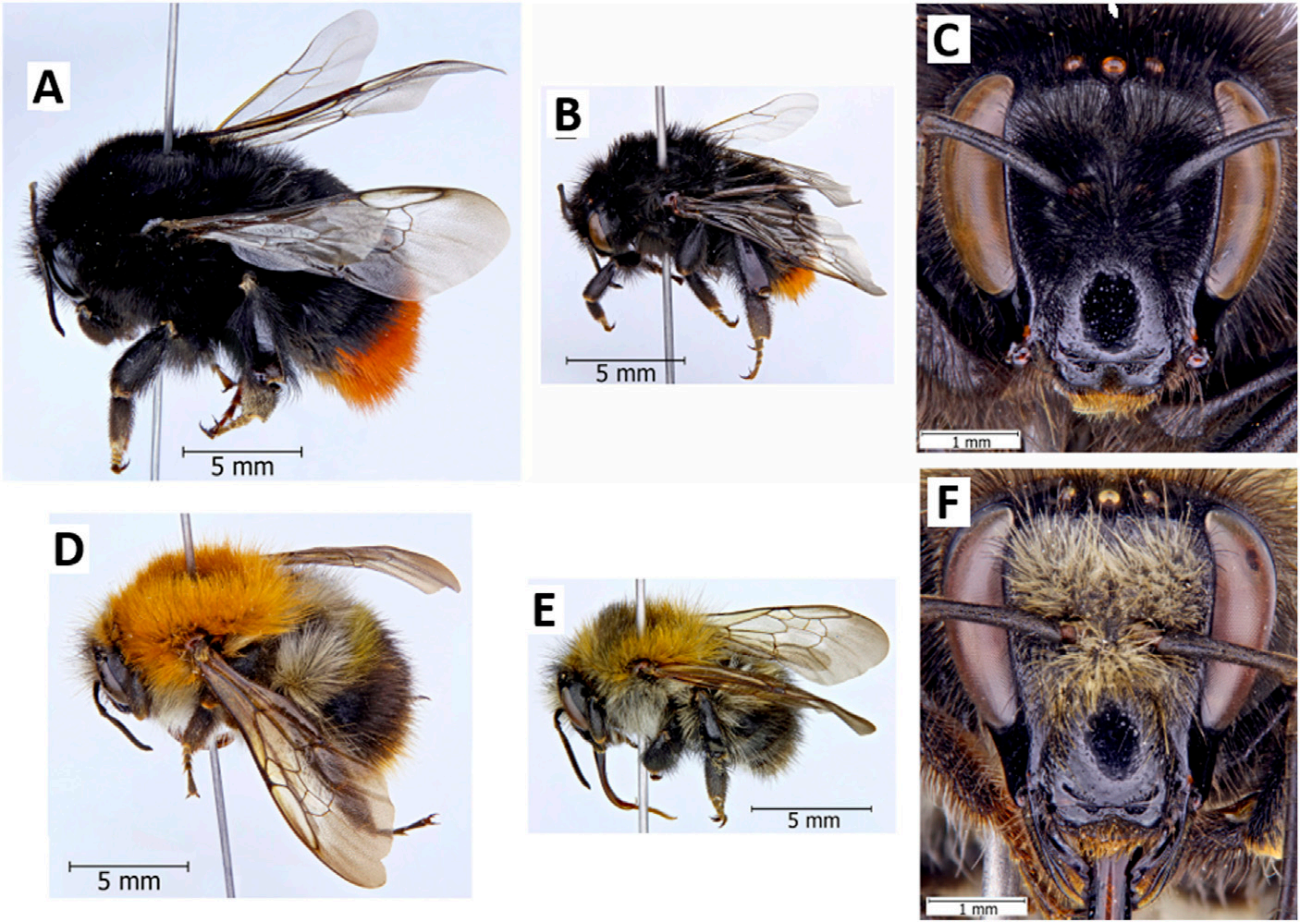
Queens and workers of *Bombus lapidarius* **(A–C)** and *B. pascuorum* **(D–F). (A)**, **(D)**—Queen; **(B)**, **(E)** Worker; **(C)**, **(F)** Head of worker in frontal view. Photo credit Vladimir G. Radchenko^4^.

*Bombus pascuorum* (Figures 1A,B) and *B. lapidarius* (Figures 1D,E) are two common and widespread bumblebee species found across Europe in a wide range of environmental conditions (Rasmont et al., 2015, 2021). Both are generalist foragers found in almost all habitats, and for which high intraspecific variation has been described (Lecocq et al., 2015, 2020; Williams et al., 2020; Rasmont et al., 2021). In terms of nesting, both species prefer relatively sheltered areas, although *B. lapidarius* usually settle underground in different cavities, while *B. pascuorum* builds its nests more often aboveground on the soil surface among the thick grass or in abandoned bird nests or tree holes (Alford, 1975; Radchenko & Pesenko, 1994). These two species were chosen as they belong to the two groups of the genus *Bombus*, the “short-faced” “pollen-storers” (*B. lapidarius*; Figure 1C) and the “long-faced” “pocket-makers” (*B. pascuorum*; Figure 1F) (Cameron et al., 2007; Goulson, 2010; Peeters et al., 2012).

**FIGURE 2:**
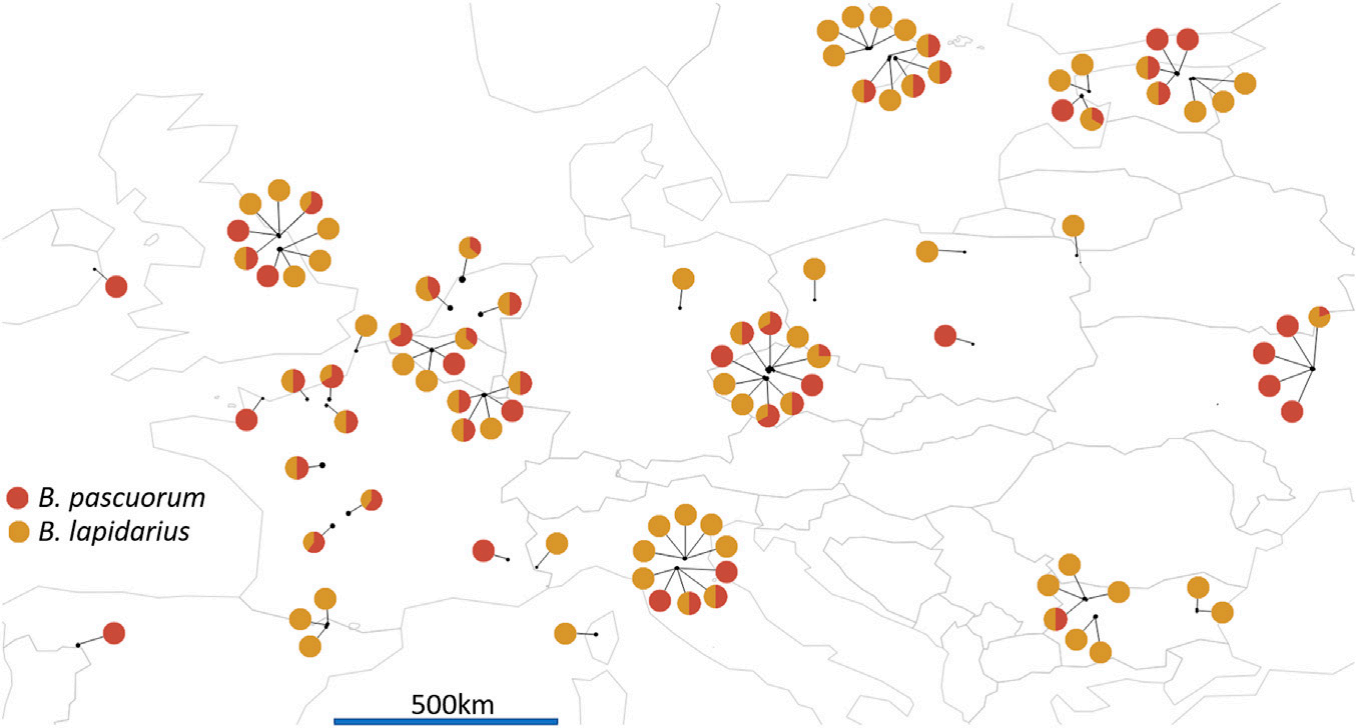
Map of the 97 sampling site locations across Europe. The pie charts represents the number of *B. pascuorum* (in red) and *B. lapidarius* (in orange) specimens sequenced per site. Data visualised was with R packages ‘rworldmap’ (South 2011) to draw the map and ‘ggplot2’ (Wickham 2009) to draw the pie charts.

Specimens were collected from 103 sampling sites in 16 countries across Europe during the bumblebee foraging seasons of 2018–2020 (Figure 2). Where possible, a paired sampling took place in both an agriculturally intensive landscape and a nearby low anthropogenic impact area (so-called natural landscape), such as forest, nature reserve, or unmanaged meadow by the same collector (see Supplementary Table S1 for sampling description and Supplementary Table S2 for full site coordinates). Most specimens were frozen and shipped on ice; although some collectors preferred to pin the recent specimens before shipment. Upon reception, specimens were immediately stored at −20°C. We selected only female specimens for further processing. After excluding 10 samples with no geographic data available, a total of 118 candidate *B. pascuorum* and 88 candidate *B. lapidarius* workers were collected, leaving 15 countries and 97 sites remaining (Table 1).

**TABLE 1:** The total number of specimens by country and species that have been RAD sequenced and remained after filtering, with the number of sampling sites in parentheses, and total numbers in italic.

	No. of specimens	
Country	*B. pascuorum*	*B. lapidarius*
Belgium	11 (8)	9 (7)
Bulgaria	8 (7)	—
Czechia	11 (9)	10 (7)
England	8 (7)	10 (4)
Estonia	10 (8)	5 (5)
France	11 (6)	15 (7)
Germany	1 (1)	—
Italy	5 (5)	3 (3)
Netherlands	13 (3)	9 (3)
Poland	3 (3)	—
Portugal	—	4 (4)
Spain	5 (3)	—
Sweden	10 (9)	3 (3)
Ukraine	1 (1)	5 (5)
***Total***	*97 (70)*	*73 (48)*

### DNA extraction

Before extraction, the abdomen of each sample was removed under sterile conditions to reduce contamination from gut contents and microbiota (Vanderplanck et al., 2019b). DNA was extracted from the remaining tissue using an Invisorb Spin Tissue Mini Kit (Invitek Molecular GmbH, Germany) according to manufacturer’s instructions. To maximise yield three changes were made to the protocol: silica sand and stainless steel beads (Qiagen) were added to the lysis step using a TissueLyser II (Qiagen), 500–600 µl RLS buffer was used to increase DNA concentration, and samples were split across multiple spin filters when possible. DNA was cleaned and concentrated using Amicon Ultra Centrifugal Filters (Merck Millipore, Belgium). Excess RNA was apparent when the DNA quality was visually assessed using agarose gels, so samples were treated with 1 µl of RNAse A (Thermo Fisher Scientific, Belgium) at 2 mg/ml per 20–30 µg of DNA. Concentration was measured using a TECAN platform and Quant-it PicoGreen dsDNA Assay Kit (Thermo Fisher Scientific, Belgium) before normalisation to 50 µl of 20 ng/μl for library preparation.

### Library preparation and RAD-Sequencing

Samples were outsourced to Floragenex Inc. (Oregon, United States) for library preparation and RAD-Sequencing. Samples underwent classic single-enzyme digestion with PstI (Baird et al., 2008), before shearing and single-end sequencing on an Illumina HiSeq 4000 with a target read length of 125 bp or 150 bp. We opted to use single-end sequencing as Jackson et al. (2018) found that it was not significantly different in reliably genotyping samples when compared with paired-end sequencing.

### Data pre-processing

Sequencing data from Floragenex underwent a series of pre-processing steps before analysis. Finding appropriate thresholds and parameters for QC and read/locus filtering can be challenging. Overconservative handling of sequencing data may exclude ‘true’ positive outlier loci, while inappropriately liberal treatments may result in the inclusion of false positives; finding a balance in settings and parameters is key to producing robust but reliable data from noise and artefacts (Shafer et al., 2015; Paris et al., 2017; O’Leary et al., 2018). To mitigate such issues in our own dataset, close attention was paid to similar literature (e.g., Theodorou et al., 2018; Jackson et al., 2020; Silva et al., 2020) and to published guidance on parameter optimisation (Andrews et al., 2016; Paris et al., 2017; Rochette and Catchen, 2017; O’Leary et al., 2018).

Firstly, residual sequencing adaptors were removed by Floragenex prior to data delivery. Afterwards, samples were demultiplexed using Stacks (2.0) *process_radtags* using options *c*, *r*, and *q*, which remove reads with uncalled bases, ‘rescue’ barcodes and RADtags, and discard reads with low quality scores, respectively. Barcode and RADtag rescue will correct errors that are within a set distance of a ‘true’ barcode or cut site (Catchen et al., 2013). After demultiplexing, reads were further trimmed for quality using Trimmomatic (0.38) with the following settings: *slidingwindow*:5:28 to ensure the average base PHRED quality never drops below 28 over a sliding window of 5 bp, *minlen*:80 to remove reads shorter than 80 bp, and finally the standard recommended *leading*:3 and *trailing*:3 to remove low quality and ambiguous base calls at the leading and trailing ends of a read (Bolger et al., 2014). The Bowtie2 (2.4.2) aligner was used to first generate indexes for the reference genomes of *Bombus opulentus* (GenBank Accession: GCA_014737405.1) and *B. pyrosoma* (GCA_014825855.1), the closest available relatives to *B. pascuorum* and *B. lapidarius,* respectively, using default settings (Landmead and Salzberg, 2012; Sun et al., 2021). After index generation, reads were aligned to the genome using end-to-end mode under default settings. The genome alignments produced by Bowtie2 require further processing before they can be used for analysis. SAMtools (1.11) was used to filter out any unmapped reads using *view*, sort the files by their genome coordinates using *sort*, and create an index for each alignment (which helps speed up read time) using *index* (Li et al., 2009). Finally, Stacks was then used to identify the SNP variants present in the dataset, using the *ref_map* wrapper under default settings.

### Quality control

VCFtools (0.1.16) was used to report the number of RAD tags (as called by Stacks *ref_map*) and average read depth for each sample using *-depth* (Danacek et al., 2011). *B. pascuorum* and *B. lapidarius* samples with fewer than 100,000 or 90,000 sites respectively, or an average depth below 10x, were removed. VCFtools was also used to check for heterozygosity using *-het*; samples with an inbreeding coefficient (*F*_IS_) higher than 0.5 were removed. Additionally, VCFtools was used to generate a list of loci with a maximum mean depth of twice the average read depth and maximum proportion of missing data of 25%. The maximum read depth is capped at twice the average to prevent paralogous regions being counted twice. Loci that failed either of these thresholds were added to a blacklist.

### Species identification and kinship analysis

After QC, libraries were initially mapped to the *B. terrestris* reference genome using Bowtie2, and run through Stacks with no blacklist, to act as a basis for species determination (Sadd et al., 2015). A combination of approaches were used to determine species, as initial identifications were not always performed by experts in the field, which can cause misidentification due to visual similarity, e.g. *B. lapidarius* being highly similar in appearance to *B. ruderarius* and *B. pascuorum* being similar to *B. humilis* and *B. muscorum* (Rasmont et al., 2021). Bioinformatic methods of species identification relied primarily on the inference of admixture coefficients in R-package sNMF run from *K* = 1:10 with 10 reps, followed by cross-entropy and admixture visualisation to identify the number of groups within the population, with additional use of relatedness indices calculated by VCFtools, *-relatedness* and *-relatedness2*, to highlight outliers. *B. pascuorum* with a *-relatedness* or *-relatedness2* result of below −0.4 were considered off-target species. For *B. lapidarius*, the thresholds to be considered off-target species were below −0.3 for *--relatedness* and below −0.5 for *-relatedness2*.

Relatives were identified by grouping samples by natural population using VCFtools *--relatedness* and *-relatedness2*. For both species, samples were considered siblings if they had a *--relatedness* result of above 0, or a *-relatedness2* result of above 0.1. Standard thresholds (Jackson et al., 2018) were made slightly stricter to avoid second-degree relationships (Manichaikul et al., 2010).

### Environmental data

CORINE land cover proportion was calculated for a 0.5 km radius around each sampling site using a custom R-script containing the following packages: rgdal 1.5–23 (Bivand et al., 2021), raster 3.4–13 (Hijmans, 2020), plyr 1.8.6 (Wickham, 2011), dplyr 1.0.7 (Wickham et al., 2021), sf 1.0–2 (Pebmesa, 2018), fasterize 1.0.3 (Ross, 2020), and data.table 1.14.0 (Dowel and Srinivasan, 2021). A radius of 0.5 km was chosen since estimates of bumblebee foraging range average at around 300m, with a maximum of 600–800 m, although this does vary with species and the method used (Osborne et al., 1999; Wolf and Moritz, 2008). In particular, the mean worker foraging distances differs significantly between species: *B. pascuorum* exhibited significantly smaller mean foraging distances (average 272 m) than *B. lapidarius* (average 536 m) (Redhead et al., 2016).

**FIGURE 3:**
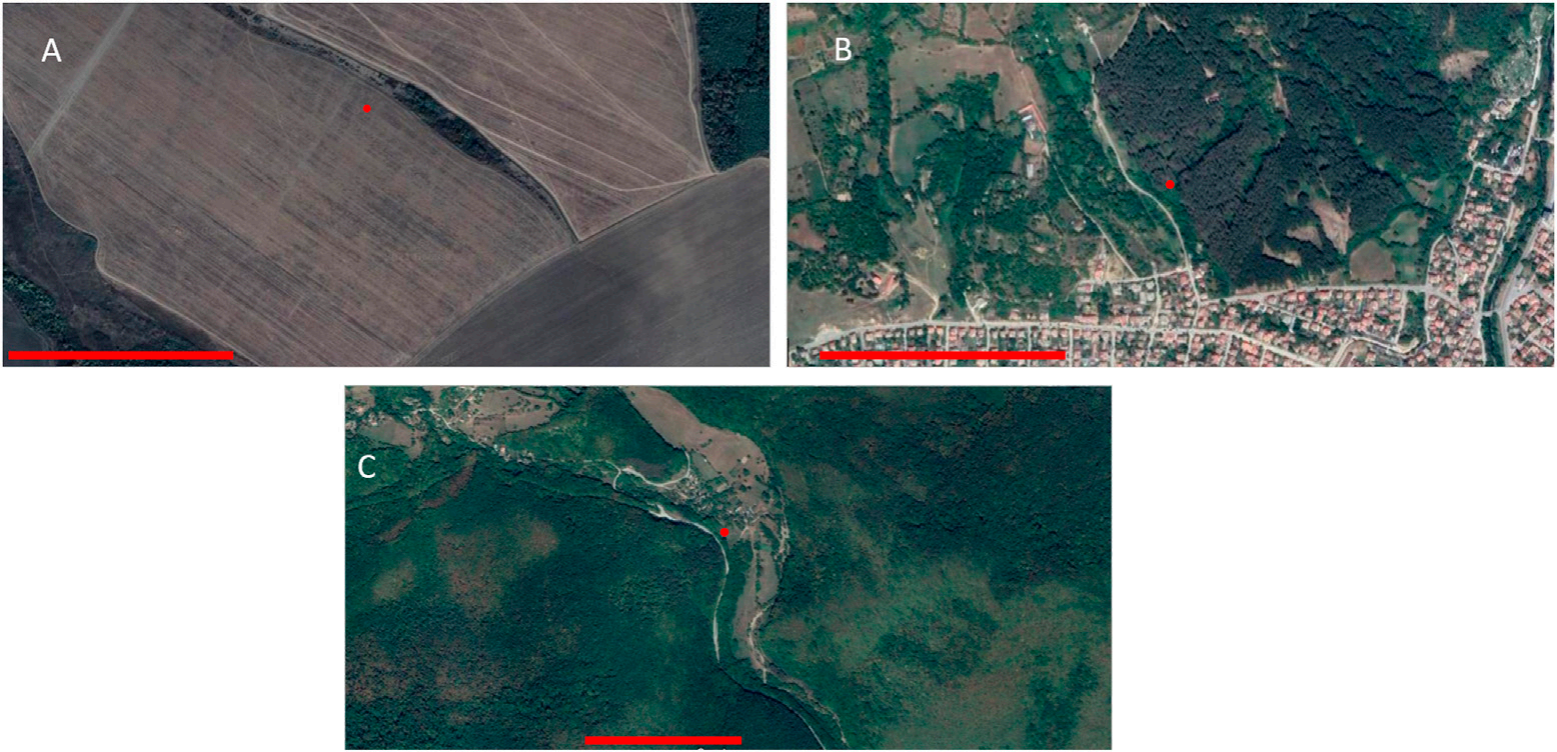
Example of sampling sites, as seen using satellite imagery (Map data: Google Maps 2022, Maxar Technologies). A set of sites in Bulgaria: **(A)** shows an area of intensive farmland, close to the south of Devene, categorised as ‘high agriculture’; **(B)** shows an ‘intermediate’ area of mixed natural and agricultural land, to the west of Etropole, while **(C)** shows the heavily forested ‘low agriculture’ area to the south of Boykovets. Scale bar represents 500 m.

CORINE data, generated in 2018, classifies land as agricultural or natural based on usage, e.g. farmland, pasture, and forest. The proportion of each sampling site belonging to agricultural, urban, or natural (after water was removed) was calculated. Sampling sites were arbitrary ordered into three categories; <33.3% (= low agricultural), 33.3–66.6% (= intermediate); and >66.6% (= high agricultural). Figure 3 illustrates the terrain of the high agricultural (Figure 3A), intermediate (Figure 3B), and the low agricultural (Figure 3C) categories. As this analysis was performed after the sampling - many of the paired sampling locations did not hold up after CORINE categorization. Analysis is not based on the initial pairs, only on the categorization following the CORINE data.

Specimens from neighbouring sites (<7 km) were grouped into populations. Populations were a minimum of 20 km apart. CORINE land cover proportions were also analysed within the 7 km radius. Only populations consisting of three or more samples were used for population genetic estimations. 20 such populations were identified, retaining 71 *B. pascuorum* (73.2% of samples) and 55 *B. lapidarius* (75.3%) (Table 2). The thresholds for division were based on the flight distances of mated queens; *B. pascuorum* and *B. lapidarius* queens fly up to three and 5 km respectively (Lepais et al., 2010), but not more than 10 km (Kraus et al., 2008).

**TABLE 2:** Grouping of all remaining specimens (after filtering) into populations, their land cover categorisation, and the number of specimens in each population, per species.

Population	Land cover category	*B. pascuorum*	*B. lapidarius*
Belgium north	High agriculture	7	5
Belgium south	High agriculture	4	4
Bulgaria north	High agriculture	3	0
Bulgaria south	Low agriculture	3	0
Czechia north	High agriculture	6	9
Czechia south	Intermediate	3	0
England Huggate	High agriculture	4	9
England Moors	Low agriculture	4	0
Estonia perressare	Low agriculture	3	0
Estonia Vinni	Intermediate	3	4
Italy rural	High agriculture	3	0
Italy wilds	Intermediate	0	3
Netherlands dog park	High agriculture	3	3
Netherlands Leiden	High agriculture	4	3
Netherlands Texel	Intermediate	6	3
Portugal	Low agriculture	0	4
Spain mountain	Low agriculture	5	0
Sweden forest	Low agriculture	6	0
Sweden uppland	Intermediate	4	3
Ukraine Kiev	Intermediate	0	5

### Population structure and isolation by distance

To explore possible interference of structure and isolation across populations, we tested for significant relationships between geographic distance and genetic divergence by Mantel testing (Mantel, 1967). Mantel testing was performed using R-package ape4 (Dray and Dufour, 2007) on the bumblebee populations described above. Mantel testing attempts to derive the strength and direction of correlation between two matrices; in our case, a matrix of pairwise genetic distances (*F*_ST_)—generated by Stacks, linearized using the formula (*F*_ST_/1-*F*_ST_), and haversine geographic distance between populations, calculated using R-package geosphere (Hijmans, 2019). The number of repetitions was set to 10,000.

### Demographic history

To infer demographic history, *dadi* version (2.1.1) was used to generate folded Site Frequency Spectra (SFS) (Gutenkunst et al., 2009). Given the absence of population structure (see the results on population structuring), we decided to here group the samples of each species into one cluster each. The VCF-file generated by Stacks:ref_map was converted into an SFS, folded, and used as a basis for 1D-demographic models of ‘growth,’ ‘bottlegrowth,’ ‘two_epoch,’ and ‘standard neutral model,’ using 100 optimisation runs. Bootstrapping was used to estimate confidence intervals for each model parameter; AIC was calculated to determine which model best fitted the available data; Likelihood Ratio Tests (LRTs) were used to compare the neutral model with the other models.

### Scans for loci under selective pressure

Three different methods were used to detect loci under selective pressure and were performed on all specimens that passed the above described QC-steps. The first genome scan method we used was BayeScan (Foll and Gaggiotti, 2008). BayeScan uses differences in allele frequencies between discrete populations and the multinomial-Dirichlet model to find evidence of local adaptation. BayeScan (2.1) was run with default settings. Secondly, BayeScEnv (a similar method to BayeScan) was used, which takes a gradient approach to populations and includes environmental variables in calculations to identify local adaptation (de Villemereuil Gandaggiotti, 2015). BayeScEnv (1.1) was run with default settings except *-nbp* which was set to 10, *-pilot* which was set to 2000, and *-burnin* which was set to 10,000. The average proportion of agricultural land cover for each of the categories, normalised to between 1 and 0, was used as environmental variable. Finally, the *lfmm* function in R-package *LEA* was used to perform regression analysis on genomic data and environmental variables to associate allele frequencies with environmental factors (Frichot and Francois, 2015). Here, land cover data was inputted as a list of agricultural land cover proportion for each sample. *Lfmm* was run with *K* = 1 (given the absence of population structure) and five repetitions. The z-scores of each run were combined, averaged (median) and adjusted for genomic inflation factor lambda; λ was set to 0.6 for *B. pascuorum* and 0.5 for *B. lapidarius* based on the histogram visualisation as directed by Francois’s LEA tutorial. *p*-values were then adjusted for multiple testing using the Benjamini-Hochberg method (Benjamini and Hochberg, 1995).

### Annotation

As both genomes used for read alignment were published recently (Sun et al., 2021), levels of genome annotation were insufficient to provide detailed functional information on the loci highlighted by previous programs. We tried to bypass these shortcomings by using a custom R-script and the package seqinr (Charif and Lobry, 2007). The reference genome sequence for 100 bp either side of a highlighted SNP was extracted and saved into a data frame, and then investigated for matches in the NCBI database, using megablast (version 2.12.0) under default settings, filtering for matches to Arthropoda exclusively (Altschul et al., 1990). BLAST results were manually searched for ‘informative’ descriptions—i.e., the name of a specific gene or function, not just a hit to an unannotated stretch of genome.

Given that bumblebees are not model organisms, performing Gene Ontology (GO) analysis is a substantial challenge. However, using BioDBnet’s dbReport function, it was possible to perform an elementary analysis on the available data (Mudunuri et al., 2009). Gene IDs (e.g., LOC100749465) were extracted from the BLAST results for each species and processed with dpReport, which searches several genomic databases and returns all available information for a given identifier. From these results, the GO data for each locus was retrieved, covering the three standard GO categories, “cellular component”, “biological process,” and “molecular function” (Ashburner et al., 2000).

To determine the likelihood of non-synonymous mutation in the highlighted SNPs, each unique locus with an informative BLAST result was investigated to determine whether the difference in major and minor alleles would have a functional consequence. For each locus, the reference sequence was extracted, but the SNP location in the reference sequence was replaced with the major and minor allele from our own sequencing data, as the reference genome and our study species sequences may differ. The major allele sequence was BLASTed (version 2.13.0), the results filtered for matches to Arthropods, and searched for the highest quality informative match. If the query sequence was matched to a coding region (CDS), ExPASy’s web ‘translate’ tool (Gasteiger et al., 2003) was used to determine the open reading frame (ORF) of the sequences, and whether there was a difference in amino acid sequence between the major and minor alleles.

## Results

### Sequencing

118 *B. pascuorum* and 88 *B. lapidarius* candidates were distributed across five lanes of Illumina HiSeq 4000 single-end sequencing. A total of 1.67 billion reads were produced across all plates with an average of 334 million per plate. After demultiplexing and initial low-quality filtering, 92.8% of reads were retained, with 4.28% dropped due to ambiguous barcodes, 2.79% dropped due to ambiguous RAD-tags, and 0.08% dropped due to low quality, leaving 1.55 billion reads remaining.

### Pre-processing, QC and kinship analysis

Individual sample libraries underwent further processing with TRIMMOMATIC. The average sample library input 3.24 million reads, and retained 2.78 million of them (85.5%). Initial species identification, found 118 candidate *B. pascuorum* and 88 candidate *B. lapidarius*, which were then re-mapped to the genomes of *B. opulentus* and *B. pyrosoma,* respectively, and resulted in an average read retention of 91.4%, and 79.2%, respectively. After the remapping, more specimens were removed due to several additional QC steps (see Supplementary Table S3), leaving 97 *B. pascuorum* and 73 *B. lapidarius* samples remaining.

### Loci filtering

Stacks identified 115,199 loci in the *B. pascuorum* and 151,037 loci in the *B. lapidarius* samples, with a mean number of 131.4 and 129.3 sites per locus respectively (Table 3). Effective per-sample coverage was 47.7x in *B. pascuorum* and 35.4x in *B. lapidarius*. After several filtering steps a total of 13,989 loci in *B. pascuorum* (12.14%) and 13,980 in *B. lapidarius* (9.26%) remained for analysis (Table 3).

**TABLE 3:** Per species locus counts for loci generated and filtered by Stacks, and blacklisted by VCFTools.

	*B. pascuorum*	*B. lapidarius*
Raw locus count	115,199	151,037
VCFTools blacklist	62,054	51,433
Percent blacklisted	53.9%	34.1%
Stacks internal filter	39,156	85,624
Percent filtered by Stacks	34.0%	56.7%
Final locus count	13,989	13,980
Percent retained from raw	12.1%	9.3%

### Land cover categories

Samples were separated into three categories based on the proportion of ‘natural’ land cover at their collection site, based on CORINE analysis (Supplementary Table S4). The *B. pascuorum* dataset contained 62 samples in the ‘high agriculture’ category, 19 samples from the ‘low agriculture’, and 16 from the ‘intermediate’ category. In the *B. lapidarius* dataset, 52 samples were classed as ‘high agriculture’, 10 as ‘low agriculture’, and 11 as ‘intermediate’ (Supplementary Table S4).

### Demographic history

Using *dadi* to test several models of demographic history, and comparing their AIC for goodness-of-fit purposes, we found that in *B. pascuorum*, the ‘growth’ model (AIC = 522.24) fit our data the best followed very closely by ‘bottlegrowth’ (AIC = 524.42; Table 4). In *B. lapidarius*, results were similar, with ‘bottlegrowth’ being the best fitted model (AIC = 358.62), followed closely by ‘growth’ (AIC = 356.64; Table 4). All three growth-based models fit the data better than the neutral model, in both species (*p* = <0.001). Models were rerun after filtering out outlier loci identified with BayeScan, BayeScEnv and LEA (see below) but were not meaningfully different (Supplementary Table S5). Populations of *B. pascuorum* increased in sizes about 70,600 years ago, while population growth for *B. lapidarius* started 98,400 years ago (Supplementary Table S6).

**TABLE 4:** Demographic model Log Likelihood (LL) and Akaike Information Criterion (AIC) generated by *dadi*. The best-fitting model for each species has been highlighted in bold. For all models, the parameter T refers to the time at which change began, in units of 1.5 N_a_ (ancestral population size) generations. Nu refers to the ratio of modern to ancient population size; NuB is the ratio of the bottleneck population size to ancient population size; NuF is also the ratio of modern to ancient population size.

Species	Model	Log likelihood	AIC	Best fit parameters
*B. pascuorum*	Neutral model	−6313.55	—	—
	**Growth**	−**259.12**	**522.24**	Nu = 22.43, T = 0.55
	Bottlegrowth	−259.21	524.42	NuB = 1.04, NuF = 22.51, T = 0.53
	Two Epoch	−374.74	753.48	Nu = 12.44, T = 0.29
*B. lapidarius*	Neutral model	−6386.24	—	—
	Growth	−176.32	356.64	Nu = 18.94, T = 0.59
	**Bottlegrowth**	−**176.31**	**358.62**	NuB = 1.01, NuF = 18.99, T = 0.58
	Two epoch	−260.75	525.50	Nu = 10.74, T = 0.33

### Population structure

In *B. pascuorum*, the average *F*_ST_ between populations was 0.103, ranging from 0.174 (Estonia Vinni—Italy Rural) to 0.058 (Belgium North—Czechia North) (Supplementary Table S7). In *B. lapidarius*, the *F*_ST_ average was 0.120, with a maximum of 0.224 (Sweden Uppland—Portugal) and a minimum of 0.053 (Ukraine—Czechia North) (Supplementary Table S8). A Mantel test was performed to compare geographic distance and genetic distance (Table 5) and with outlier loci removed (Supplementary Table S9). In *B. pascuorum*, the correlation between geographic and genetic distance was moderate, *r* = 0.35, and significant, *p* = 0.004. In *B. lapidarius* the correlation was also moderate (*r* = 0.39) but not significant (*p* = 0.058). sNMF clustering analysis was also performed on the full dataset (including samples that did not fall into populations) and found *K* = 1 as the most likely scenario for both *B. pascuorum* and *B. lapidarius* (Supplementary Table S10). Overall these results indicate a general lack of population structure across Europe for both species, with mild isolation by distance in *B. pascuorum*.

**TABLE 5:** Mantel test results for both species, comparing geographic distance (Haversine distance in km) against genetic distance (*F*_ST_ calculated by Stacks).

Species	r	*P*	Std. Obs	Expectation	Variance
*B. pascuorum*	0.353	0.004	23067	−0.0028	0.0238
*B. lapidarius*	0.392	0.058	16064	0.0002	0.0595

### Outlier detection

Across all programs, we detected 191 unique loci under selective pressure in the *B. pascuorum* specimens, and 260 unique loci in the *B. lapidarius* specimens (Supplementary Tables S11, S12). LEA detected 179 loci in *B. pascuorum*, and 196 in *B. lapidarius*, BayeScan found 12 and 55 loci, and BayeScEnv three and 16, respectively. In *B. pascuorum*, all three loci detected by BayeScEnv were also highlighted by BayeScan, while in *B. lapidarius*, only seven were common to both programs. No overlap was found between LEA and BayeScan or BayeScEnv.

### Annotation results

To better understand the function of the genes under selective pressure, sequences associated with each SNP highlighted by BayeScan, BayeScEnv, and LEA were BLASTed for similar sequences (Supplementary Tables S13, S14). The majority of hits were matching uninformative sequences, so results were manually studied for functional information. *B. pascuorum* had informative hits for 26 or 13.6% of loci, while *B. lapidarius* had hits for 53 loci or 19.2% (Supplementary Tables S13, S14).

In terms of GO analysis, very few queries (loci with gene IDs such as LOC100749465; unique loci may have multiple queries) returned terms (Supplementary Table S15). For *B. pascuorum*, 26 unique loci with informative results lead to 97 input queries, of which 13 (13.4%) returned “Cellular Component” descriptors, 6 (6.2%) returned “Biological Process” descriptors, and 16 (16.5%) returned “Molecular Function” descriptors (Supplementary Table S15). The most common “Cellular Component” category was GO:0005634 (nucleus); three “Biological Process” categories showed equal distribution (GO:0006629 lipid metabolic process, GO:0006397 mRNA processing, GO:0006139 nucleobase-containing metabolic process); and “Molecular Function” had three categories tie for most common with GO:0003677 DNA binding, GO:0005524 ATP binding, and GO:0003723 RNA binding. For *B. lapidarius*, 53 unique loci with informative results generating in 337 queries, of which 36 (10.7%) had “Cellular Component” descriptors, 26 (7.7%) had “Biological Process” descriptors, and 42 (12.5%) had “Molecular Function” descriptors (Supplementary Table S15). The most common “Cellular Component” category was GO:0016021 integral component of membrane; for “Biological Process”, GO:0005975 carbohydrate metabolism and GO:0051301 cell division were both most common; Five categories for “Molecular Function” showed equally high prevalence: GO:0005524 ATP binding, GO:0003677 DNA binding, GO:0046872 Metal ion binding, GO:0046983 Protein dimerisation activity, and GO:0003700 Sequence-specific DNA binding/transcription factor activity.

The same unique loci were studied to find evidence of non-synonymous mutations. In *B. pascuorum*, 19.23% of the loci investigated were found to have non-synonymous differences between the major and minor alleles present in the sequencing data; for *B. lapidarius*, the percentage was 26.42 (Supplementary Table S16). Of the non-synonymous SNPs in coding regions, several associated genes with interesting functions were identified (Supplementary Table S16); In *B. pascuorum*, transcriptional regulators (such as an ATRX homolog), lipid metabolism-related enzymes (such as triacylglycerol lipase), titin (an important muscle structure protein), and neurodevelopment proteins (such as notch) were prominent. In *B. lapidarius*, genes highlighted include several neurodevelopment and behaviour-related proteins (such as ataxin, SOX, and tyrosine-protein phosphatase 99A-like), as well as transcription factors (Forkhead transcription factor FD4), and TipE, a sodium-channel structural protein with strong associations with insecticide resistance.

## Discussion

### Demographic history

From our demographic analysis we found that the ‘growth’ and ‘bottlegrowth’ were the best models for our data of *B. pascuorum* and *B. lapidarius*, respectively. In both cases, the other growth model—i.e ‘bottlegrowth’ for *B. pascuorum* and ‘growth’ for *B. lapidarius*—also fit the data incredibly closely and was not far behind in terms of log likelihood and AIC. This pattern is similar to that of another study detecting clear signals of past population growth for *B. lapidarius*; with ‘growth’ as best fitting model, followed closely by ‘bottlegrowth’ (Theodorou et al., 2018). Studies focused on understanding the evolution and demographic history of both *B. pascuorum* and *B. lapidarius* indicate that both species likely retreated to various ‘refugia’ across Europe during the last glaciation period (Pirounakis et al., 1998; Widmer and Schmid-Hempel, 1999; Lecocq et al., 2013). The last glaciation period is estimated to have taken place between 110,000—12,500 years ago; our own calculations suggest that growth began approximately 70,600 years ago for *B. pascuorum* and 98,400 years ago for *B. lapidarius*. Overall, our results are congruent with the idea that both species had smaller populations, likely during the ice age as other studies have indicated, and have since grown considerably (Theodorou et al., 2018).

### Population structure and isolation by distance

Our sNMF results indicated a lack of significant population structure across Europe for both species. This is in line with previous research using microsatellite markers detecting no or slight population structuring for the bumblebees on the local and even continental scale (Dreier et al., 2014; Maebe et al., 2015). The only population of *B. pascuorum* in our dataset that occurred beneath the Alps (Italy_wilds) showed much higher *F*_ST_-values compared to the other pairwise estimates. This result might correspond to the two described gene pools of *B. pascuorum* that are separated by the Alps as suggested by Widmer and Schmid-Hempel. (1999). Of the 4 *B. lapidarius* subspecies that are described in Europe, two are relevant to our sampling; *B.l. lapidarius* from the European mainland, and *B.l. decipiens*, from the Iberian Peninsula (Lecocq et al., 2013). Although sNMF did not suggest a second population, the pairwise *F*_ST_ of samples from Portugal was considerably higher than average (0.111 without Portuguese samples), suggesting a pattern of differentiation similar to the Iberian subspecies (Lecocq et al., 2013, 2020). Mantel testing results found mild but significant IBD in *B. pascuorum* but not in *B. lapidarius*, implying strong gene flow between populations, and agreeing with the sNMF results. Dreier et al. (2014) found similar results using microsatellite markers; weak but significant IBD in *B. pascuorum* and insignificant results in *B. lapidarius*. Theodorou et al. (2018) also found a lack of IBD in *B. lapidarius*, which contrasts with the weak but significant IBD across Europe described for *B. lapidarius* (Lecocq et al., 2013, 2017; Theodorou et al., 2018). Overall, these mixed results paint a similar picture; weak IBD is possibly present in both species but strength and presence vary by sampling and method.

### Local adaptation

By comparing bumblebees from natural, agricultural, and intermediate areas we were able to find genes associated to agriculture over a large geographic range within two bumblebee species. Furthermore, we were able to exclude the population differentiation from neutral population structuring or historical geographic isolation. In general, this main result implies that bees of agricultural areas differ genetically from bees from more natural areas. That bees of agricultural areas tend to experience different strengths of certain pressures than bees from natural areas would be logical given the difference in, among others things, pesticide use, habitat fragmentation, and availability of food resources between these different habitats (e.g., Goulson, 2010; Cameron and Sadd, 2020). However, in our study, we detected no population structuring at putatively neutral loci for both species across a large part of their distribution in Europe. With this in mind, one might think that due to the likely high levels of gene flow supporting the lack of differentiation on a continental scale, the observed differences may not appear between bees from agricultural and natural locations, and especially not between locations within such close proximities (<50 km). However, that we were still able to detect outlier loci here, suggests that these loci have been selected and maintained in agricultural areas.

Although we were able to associate genes with agricultural stressors, we cannot exclude that these genes might also be correlated with other causes of (anthropogenic) stress, such as: urbanization. Indeed, urbanization can stress bees in a similar way to agriculture. This includes heat stress due to the heat island effect (e.g., Chapman et al., 2017; Levermore et al., 2018), nutritional stress due to decrease in floral diversity, disturbance of bee foraging patterns, and increased impervious surface and habitat fragmentation (e.g., Fortel et al., 2014; Zhao et al., 2016), and chemical stress through all kinds of environmental pollution (e.g., Hladun et al., 2015; Sivakoff and Gardiner, 2017; Grubisic et al., 2018). Furthermore, previous research found several of the same gene ontology categories as outlier loci being correlated to urbanization (Theodorou et al., 2018) or to a bioclimatic landscape (Jackson et al., 2018, 2020). Further work/analysis needs to be done on other features of anthropogenic land use to exclude them as contributors to the patterns we observed.

### Primary candidate genes

Based on the BLAST search, we were able to link some of the detected candidate genes to ongoing agricultural stressors. In both *B. pascuorum* and *B. lapidarius*, we detected several SNPs in the coding regions of genes (some synonymous and some not) linked with neurodevelopment, transcriptional regulation, and metabolism, as well as genes related to muscle development and lipid metabolism in *B. pascuorum*, and genes implicated in detoxification and cell signalling in *B. lapidarius* (Supplementary Tables S13-S16). To us, these would be the primary candidate genes for further investigation or validation. Although the presence of non-synonymous differences between the major and minor alleles provides the strongest evidence of selection, silent mutations, such as those we detected in titin in *B. pascuorum* and TipE in *B. lapidarius* should not be discounted; synonymous mutations can, for example, greatly affect codon usage/bias, mRNA splicing, and mRNA stability, with tangible consequences for gene expression (Hunt et al., 2014). The presence of SNPs in muscle-related and lipid-metabolism related genes could be particularly interesting as several studies found a strong correlation between levels of agricultural intensification and larger body size in bumblebees (Gérard et al., 2020, 2021; Nooten and Rehan, 2020). The association of genes which may relate to body size changes in our genetic data may indicate corroboration of these findings. One hypothesis is that larger-bodied bumblebees have the ability to forage at greater distances, thus overcoming habitat fragmentation (but see Gérard et al., 2020; 2021); the detection of selective pressure on titin in *B. pascuorum* may be related to muscle changes resulting from the need to fly greater distances, also related to habitat fragmentation; further study is necessary to find empirical evidence to support this. Both species had genes highlighted which are related to the nervous system and neurodevelopment, such as ataxin and notch, which may have some significance in localised behaviour, such as optimising landscape-specific foraging patterns. Ataxin has been related to circadian rhythms in *D. melanogaster* (Lee et al., 2017); Notch is involved in a range of neurogenic and regulatory processes, also in *D. melanogaster* (Lehmann et al., 1983; Xu et al., 1990; Lai, 2004). Together these may add up to complex behavioural changes in response to the environment. Additionally, in *B. lapidarius* we found associations with the heat stress and detoxification-related gene TipE. TipE, a sodium-channel structural protein has been linked to pyrethroid resistance (Warmke et al., 1997; Derst et al., 2006; Silver et al., 2014). Heat stress related genes were found to be upregulated in the face of pesticide exposure in *Spodoptera* and *Nilaparvata* (You et al., 2016; Tarnawska et al., 2019), and also identified as under selective pressure across an urbanisation gradient (Theodorou et al., 2018). This result might imply the need for *B. lapidarius* to adapt to temperature differences in natural and agricultural landscapes, or an alternative mechanism for xenobiotic resistance. Given the wide disparity in expected use of pesticides between natural and agricultural areas, it is not surprising that detoxification-related genes could be associated with agricultural-based selective pressure.

### Genome assemblies and annotation

At the time of analysis, no reference genomes for *B. pascuorum* and *B. lapidarius* were available, only *de novo* assembled genomes for relatives in the same subgenus (*Thoracobombus* and *Melanobombus,* respectively) (Sun et al., 2021). While alignment of reads to closer relatives than the initial mapping to *B. terrestris* resulted in a considerable increase in the proportion of reads that mapped to the genome and number of loci detected, bumblebee genomes have been shown to have considerable rearrangements and even differing chromosome numbers within subgenera (Owen et al., 1995; Sun et al., 2021). Thus, having species-specific high-quality reference genomes may allow for the detection of loci which are not present in close relatives. Similarly, in the absence of manual and comprehensive annotation data, many loci in exons detected in outlier analysis will be overlooked in favour of loci with concrete functional labels. Indeed, a high proportion of SNPs were present in non-coding or unannotated regions (∼81% in both species). While high-quality, curated annotation data is available for several *Bombus* species, the genus is still relatively understudied compared to other model insect species such as *Drosophila melanogaster*, and consequently the annotation data is comparatively more limited. Consequently, our Gene Ontology investigations suffer from the same data limitations. With incomplete genome annotation and no available GO annotation services for *Bombus* or better-studied close relative *Apis mellifera*, the results of the limited GO analysis were inconclusive at best, with seemingly no obvious patterns. Likewise, Gene Set Enrichment Analysis (GSEA), which looks for over- and under-representation of functions in lists of genes, cannot be performed without a reference dataset. With further study of *Bombus* and related species, sufficient GO annotation and GSEA databases will allow for understanding overall patterns and themes present in such datasets with statistical support. Because of our reduced-representation approach, certainly not all nor the strongest associations of genomic regions and loci related to agricultural stress were detected for both species. Although retrieving the genome of both bumblebee species and additional annotation data would have increased the power of this, and will increase that of other future genomic studies, they were not necessary for the outcome of our study. Indeed, we were able to reach the major target of this study which was to find any loci as signals of adaptation to agricultural stress, in both bumblebee species.

### Possible caveats

In this study, we explored and ruled out population differentiation and demographic history as interfering factors when testing for signatures of selection. Most outlier tests will identify loci as under selection when having higher (divergent selection) or lower (balancing selection) *F*_ST_-values than expected under the neutral model (Foll and Gaggiotti, 2008; de Villemereuil et al., 2014; Lotterhos and Whitlock, 2014). When significant population structuring or isolation by distance is present, this will conflict with the assumption of such neutral model (Foll and Gaggiotti, 2008; de Villemereuil et al., 2014; Lotterhos and Whitlock, 2014). Therefore, it is crucial to identify a species genetic structure to account for possible risk of identifying false positives or negatives when attempting to identify adaptive loci (as reviewed by Ahrens et al., 2018). This is why most outlier loci detection approaches already require *a priori* definition of genetic structure to inform calculations and probability tests (e.g., de Villemereuil and Gaggiotti, 2015; Frichot and Francois., 2015; Ahrens et al., 2018). In a similar manner, some approaches also account for species demographic history, such as population contraction or expansion, which can affect the species’ genetic structure and may thus also result in false positives (Lotterhos and Whitlock, 2014). In our study, we combined three different programs, which are mainly based on two different approaches: two *F*_ST_-based methods, one with and one without a direct association to an environmental parameter (BayeScEnv; de Villemereuil and Gaggiotti, 2015 and BayeScan; Foll & Gaggiotti, 2008, respectively), and an approach using latent factor mixed models (LEA) to test for the correlations between allele counts and an environmental variable while accounting for demographic structure using K latent factors (Frichot and Francois, 2015). Combining such outlier scanning approaches increases the chance of detecting candidate loci while reducing the false positive rate (François et al., 2016). Unfortunately, in our results, only a few loci were confirmed between the *F*_ST_-based methods, while none were cross-validated with LEA. This does not mean that the detected loci are false-positives. The separate results of each of these outlier scanning approaches are useful and may differ due to the methods behind them, for instance, most of our identified loci were detected with LEA, which accounted for demographic structure. In addition, the credibility of the identified genes could be strengthened by some kind of validation, for instance by functional validation through RNAi knockout, or by performing comparative studies across bumblebee species, to determine whether these identified candidate genes are actually involved in local adaptation. This would be an important avenue for future research.

A second potential caveat to our study may be the rather limited population sizes (n≥3). While performing a population genetic study with populations represented by only three specimens was previously and usually considered too small, recent studies have shown that accurate population parameters such as *F*st-values or genetic diversity measurements can be obtained when a large number of SNPs are used to compensate for such low population sizes (Nazareno et al., 2017; McLaughlin and Winker, 2020). Here, by conducting a genomic study with approximately 14,000 SNPs per species after filtering, we met the latter criteria. Therefore, we do not consider population size to be a major issue in our study. In line with this, we would nevertheless like to emphasize that we could have chosen to RAD sequence more specimens per location, but that would have reduced the strength of our current sampling which focused on specimens sampled from multiple locations across a large geographic range in Europe.

Another possible caveat, which is related to previous one, is the difference in number of specimens per land cover category. The overrepresentation of specimens in the high agricultural category might have biased the detection of outlier loci. However, and as stated before, the combined use of different methods and approaches to identify loci under environmentally-associated selection pressure should have not excluded but reduced the impact of such bias.

### Importance for conservation and management

Understanding the precise consequences of each environmental stressor to bees would be key to preventing, mitigating, or reversing anthropogenic population declines. The aim of this study was therefore to improve our understanding of anthropogenic influences on European bumblebees by searching for genomic evidence of adaptation. Such an understanding of the loci under selection and their functions may help pinpoint the exact variations in such populations which convey advantages. The preservation of such adaptive genomic variation might then be key for resilience to anthropogenic stressors, as was discussed by Jackson et al. (2020). Furthermore, understanding which traits are necessary for positive population trends in specific environmental conditions will provide insights into which populations are more or less vulnerable to anthropogenic stressors. Thus, identifying whether genetic adaptations are present or absent within populations will allow conservationists to allocate attention and conservation resources accordingly. This is particularly important for ecological and economical key pollinators like bumblebees (Klein et al., 2007; Garibaldi et al., 2013).

## Conclusion

Through the RADSeq of 97 *B. pascuorum* and 73 *B. lapidarius,* from sites across a European gradient of agricultural-to-natural landscapes, we were able to discover signs of local adaptation and selective pressure exerted by associated stressors. Through the use of BLAST, we associated several candidate genes involved in neurological development, muscle structure, and detoxification, among others. Further study, such as whole genome sequencing, using better genome annotation, or functional studies, would first allow for the validation of these candidate genes, and when confirmed, will also help: 1) to define the identity of the drivers of selection, 2) to understand precisely how variance in these genes conveys adaptation to different environments, and 2) how this information be applied for conservation purposes.

## Data Availability

The datasets presented in this study can be found in online repositories. The names of the repository/repositories and accession number(s) can be found below: https://doi.org/10.5061/dryad.00000005j, https://github.com/AlexFHart/RADSeq-Pipeline.
